# Differential RNAi efficacy of siRNA and dsRNA targeting key genes for pest control in *Spodoptera litura*

**DOI:** 10.3389/finsc.2025.1574585

**Published:** 2025-04-30

**Authors:** Yu-Chun Lin, Yun-Heng Lu, Yun Liu, Yu-Juan Su, Yu-Hsien Lin, Yueh-Lung Wu

**Affiliations:** Department of Entomology, National Taiwan University, Taipei, Taiwan

**Keywords:** RNA interference, Mesh, IAP, *Spodoptera litura*, agricultural pest, biopesticides, Dicer-2

## Abstract

RNA interference (RNAi) is a promising gene-silencing technique for pest control that targets essential genes. We assessed the potential of double-stranded RNA (dsRNA) and small interfering RNA (siRNA) to silence *mesh* or *iap* genes in the midguts of *Spodoptera litura* larvae. Despite the theoretical promise of RNAi approaches, our findings revealed that dsRNA did not induce significant gene silencing or impact larval growth, whereas siRNA exhibited clear insecticidal effects, likely by disrupting intestinal osmoregulation and impairing larval fitness. Detailed analysis indicated that dsRNA could not be efficiently converted into functional siRNA in the midguts of *S. litura*, possibly due to the low expression levels of Dicer-2 and the rapid degradation of dsRNA within the gut environment. Furthermore, while dsRNA demonstrated greater environmental stability than siRNA under soil conditions, the inability of *S. litura* to process dsRNA effectively limits its viability as a pest control agent. These findings indicate the critical role of Dicer-2 in RNAi-mediated gene silencing and highlight the challenges and limitations of employing dsRNA-based genetic pesticides in lepidopteran species.

## Introduction

*Spodoptera litura*, a highly destructive and widespread agricultural pest, poses a significant threat to several economically important crops, including species from families such as Araceae, Poaceae, Fabaceae, Rosaceae, and Cucurbitaceae. The larvae of *Spodoptera litura*, particularly in their nocturnal feeding phases, cause substantial crop damage due to their increasing dietary demands as they mature (1, 2). Traditional pest control strategies rely predominantly on chemical pesticides, which are economically efficient but present serious environmental and human health hazards (3, 4). Consequently, there is a growing interest in sustainable and biologically-based alternatives, such as RNA interference (RNAi) (5). RNAi technology, which functions by silencing essential genes in targeted organisms, has gained attention as a novel approach to pest management. It operates through post-transcriptional gene silencing by degrading homologous mRNA, thus providing a powerful tool for the development of genetic pesticides. In recent years, RNAi has been successfully implemented in European and American agriculture, exemplified by Monsanto’s DvSnf7 genetically modified crops, which employ dsRNA to control *Diabrotica virgifera* (6). However, despite these advancements, the efficacy of dsRNA-based pesticides in controlling lepidopteran pests, such as *S. litura*, remains unclear.

The RNAi mechanism comprises several crucial steps, including dsRNA uptake, its processing into siRNAs by Dicer-2, the formation of the RNA-induced silencing complex (RISC), and the subsequent degradation of target mRNAs (7, 8). While this pathway has proven highly effective in certain insect orders, such as Coleoptera, lepidopteran species exhibit markedly lower RNAi efficacy. A primary reason for this reduced effectiveness is the inefficient conversion of dsRNA into functional siRNA within the midgut, a process that is largely regulated by Dicer-2 (9). In this study, we aimed to elucidate the molecular mechanisms contributing to the impaired RNAi response in *S. litura*, with a particular focus on the conversion of dsRNA into functional siRNA in the midgut. Our preliminary results suggest that dsRNA fails to trigger effective gene silencing in *S. litura* due to rapid degradation in the gut environment and significantly reduced expression of Dicer-2, an enzyme critical for dsRNA processing into siRNA. We hypothesize that these two factors—the hostile gut environment and insufficient Dicer-2 levels—represent the main barriers to the successful application of RNAi-based pest control in *S. litura*. To validate this hypothesis, we conducted a series of experiments, including northern blot analyses to investigate the stability and conversion of dsRNA into siRNA, as well as qRT-PCR assays to quantify Dicer-2 expression levels in the midguts of *S. litura*. Furthermore, we compared the degradation rates of dsRNA in the gut environments of *S. litura* and honey bees to gain deeper insights into the molecular and environmental constraints limiting RNAi efficacy in lepidopteran species. Gene *mesh*, which supports the epidermal cell adhesion, was used as RNAi target in the present study. The transmembrane protein Mesh is a septate-junction protein, was previously proved to induce cell-cell adhesion in *Drosophila* S2 cells (10). Mesh protein also stabilized gut microbiome in *Aedes aegypti* (11). In turn, we assume that targeting *mesh* could reduce the integrity and normal function of larval digestive system and causing mortality.

Our findings indicate that the low expression of Dicer-2, coupled with the rapid degradation of dsRNA in the gut environment of *S. litura*, significantly impedes the conversion of dsRNA into functional siRNA, thereby rendering dsRNA-based pest control strategies ineffective in this species. These results highlight the importance of further research aimed at improving dsRNA stability and enhancing conversion efficiency in lepidopterans, while also suggesting that siRNA, rather than dsRNA, may offer a more effective approach for pest management in *S. litura* and related species.

## Materials and methods

### Maintenance of *S. litura*

*S. litura*, commonly known as tobacco cutworm, was obtained from a specialized farm in New Taipei City, Taiwan. This strain was bred under laboratory conditions for multiple generations. An artificial diet was prepared as follows to meet the nutritional requirements: a) A blend was produced by mixing 90 g of kidney bean powder, 36 g of yeast extract, 33 g of wheat germ powder, and 480 mL of sterile water for 5 min. b) Subsequently, 0.036 g L-cysteine (PanReac AppliChem), 3.6 g L-ascorbic acid (PanReac AppliChem), and 0.225 g streptomycin sulfate (Bioshop) were dissolved in 60 mL sterile water. c) The solution from step b) was added to the mixture from step a) and thoroughly mixed. d) Furthermore, 9.9 g of agar was completely dissolved in 300 mL of water by boiling. e) Next, the solution from step d) was added to the mixture from step c) and homogenized for 5 min. The resulting mixture was poured into a container, allowed to solidify, and stored at 4°C until further use. Next, tobacco cutworms were placed in round transparent plastic containers measuring 8 cm (L) × 5.5 cm (H). Environmental conditions were maintained at 26 ± 1°C with a 12-h light and 12-h dark cycle. The diet was replenished daily to ensure optimal nutritional status (12, 13).

### Total RNA extraction and cDNA synthesis

Total RNA was isolated from various larval instars using TRIzol reagent (Invitrogen), following the manufacturer’s instructions. A NanoDrop OneC spectrophotometer (Thermo Fisher Scientific) was used to determine the RNA concentration. cDNA was synthesized from 500 ng of total RNA using the PrimeScript RT Reagent Kit (TaKaRa) following the manufacturer’s instructions. cDNA was diluted 10-fold for subsequent experiments (14).

### dsRNA and siRNA syntheses

Double-stranded RNA (dsRNA) targeting the *mesh* and *iap* genes was synthesized using gene-specific primers designed via the Primer-BLAST tool (NCBI). The primer sequences for *Mesh* were 5’-CGC TTC TCA AGT GTG GGT CT-3’ (forward) and 5’-TGA CCT GAC CGT GGA TGT TG-3’ (reverse), while those for *iap* were 5’-CCG TGG AGA TGA AGT CCG TT-3’ (forward) and 5’-CCC AGG GTA CGT CAT GGT TC-3’ (reverse). A *mesh* or *iap* gene fragment was PCR-amplified using “Fermentas” under the following conditions: 5 min at 96°C; 40 cycles at 96°C for 30 s, 55°C for 30 s, and 72°C for 20 s; and a final extension at 72°C for 3 min. Subsequently, the PCR product was used as a template for the second round of PCR to add T7 promoter sequences to both ends. A GenepHlow gel/PCR kit (Geneaid) was used to purify the resulting product. The MEGAscript T7 Kit (Invitrogen) was used to synthesize dsRNA against the *mesh* or *iap* genes, following the manufacturer’s instructions. TURBO DNase digestion removed the template DNA, and dsRNA was recovered using TRIzol reagent. The quality and quantity of dsRNA were assessed by electrophoresis on a 1% agarose gel and spectrophotometry. MDBio, Inc. Taiwan conducted a *mesh* or *iap* gene sequence analysis for siRNA synthesis and further predicted and synthesized three siRNA sequences.

### Gene expression analysis using real-time qRT-PCR

The expression levels of genes involved in the siRNA pathway were quantitatively assessed in each group using the SensiFAST™ SYBR^®^ Hi-ROX Kit (Bioline). The primer sequences used are listed in **Table 1**. Real-time qRT-PCR was conducted on an ABI StepOnePlus™ real-time system (Applied Biosystems) with the following protocol: initial denaturation at 95°C for 20 seconds, followed by 40 cycles of 95°C for 3 seconds and 59°C for 30 seconds. A melting curve analysis was then performed according to the manufacturer’s instructions (15, 16). Data was analyzed by ΔΔCT methods (17). Gene expression was normalized to *Actin* (NCBI accession no. OM867339) or *18S* (NCBI accession no. JX041463.1) (18, 19).

**Table 1:** The primer of candidate genes for analysis gene expression.

Primer name	Sequence
*18S-rRNA*	F: 5’CGGCGACGCATCTTTCA3’ R: 5’GCGCAGAACCTACCATCGA3’
*Dicer2*	F: 5’ TGCAGTTCTACCGAAGCCTG3’ R: 5’ GGGTTGGAGAAGGGTTAGGC3’
*Ago2-X1*	F: 5’ ACCGACTCACCGTTAGGGTT3’ R: 5’ ATGATCCGATGCGGCTTGAA3’
*Ago2-X2*	F: 5’ TGCAGCAACACAGGCAAGAG3’ R: 5’ AGGAACTGTCCCTCGGAGAT3’
*DRD2*	F: 5’ CCCGGGACTTTAACGAGGAG3’ R: 5’ GCAATGAGTGCGAGGTGGTA3’
*Actin*	F: 5’ CCCTAAGGCCAACAGGGAGA 3’ R: 5’ GACCAGAGGCGTACAGAGAG 3’

### Measurement of the mortality rate

Second-instar larvae (*n* = 15–20 per treatment, 3–5 replicates) were placed in plastic containers and starved for 12–24 h before the experiment. To ensure uniform intake, the artificial diet was replaced daily with freshly prepared feed containing dsRNA or siRNA. Each day, 3 µg of dsRNA or siRNA was added to approximately 100 mg of the artificial diet for every 10 larvae, and this feeding regimen was maintained for 4 days. Although the exact amount consumed by each larva could not be directly measured, daily diet replacement and uniform distribution minimized intake variation among larvae. After 4 days of dsRNA or siRNA feeding, larvae were provided with sufficient artificial diet and rearing space. Larval mortality was recorded daily for up to 14 days (16).

### Northern blotting

Total small RNAs were extracted from *S. litura* using a mirVana™ miRNA isolation kit (Ambion) according to the manufacturer’s instructions. For northern blot analysis of small RNAs, 1 µg of the sample was fractionated by 15% denaturing polyacrylamide gel electrophoresis (PAGE) (acrylamide:bis ratio, 19:1) containing 8 M urea in 0.5× TBE buffer. Small RNA was extracted from the midgut at various time points (2, 4, 12, and 24 hours post-feeding) following dsRNA ingestion to analyze the conversion of dsRNA into siRNA. The synthesized dsRNA was directly run on a gel as a control without undergoing the feeding process. RNAs were transferred to a Hybond-N+ nylon transfer membrane (GE Healthcare) via electroblotting and UV cross-linking. RNA oligonucleotides carrying the reverse complementary sequence of the candidate MESH siRNA were end-labeled with DIG (MD Bio, TW) to achieve high specific activity. Hybridization and washing were performed using DIG hybridization buffer according to the manufacturer’s instructions (Roche). The sequence of the probes used in northern blot analysis was as follows: MESH siRNA (5′-GAACAGATAGTCTAAACACTGGG-3′) (20).

### Protein extraction and western blot analysis

Total protein from the dissected midgut of *S. litura* was extracted by homogenization in 60 µL 1× RIPA solution (Bio Basic Inc., Ontario, Canada), followed by 20 min of ice incubation and centrifugation at 1,000 ×g for 10 min to remove debris. The samples (20 µL each) were mixed with an equal volume of 4× Laemmli Sample Buffer (Bio-Rad, Hercules, California, U.S.) containing 10% 2-mercaptoethanol. The samples were subsequently incubated at 99°C for 10 min and stored at −20°C until use. Electrophoresis was performed using the Mini-PROTEIN Tetra System (Bio-Rad) and PowerPac Basic (Bio-Rad). Protein samples (20 µL) and 5 µL of AccuRuller RGB Pre-stained Protein Ladders (Omics Bio, Taiwan) were resolved on a 10% sodium dodecyl sulfate-polyacrylamide gel and transferred onto an Immobilon Transfer Membrane (Millipore, Massachusetts, U.S.). The membrane was blocked with 5% bovine serum albumin (UniRegion Bio-Tech, Taiwan) in Tris-buffered saline with 0.1% Tween 20 (VWR Life Science AMRESCO, Pennsylvania, U.S.) and subsequently incubated with rabbit polyclonal anti-Dicer-2 (1:5000; Abcam ab4732) and mouse monoclonal actin primary antibodies (1:2500; Yao-Hung Biotechnology Inc., Taiwan) overnight at 4°C. This was followed by a 1-h incubation with horseradish peroxidase-conjugated goat anti-rabbit secondary antibody (1:1000; Millipore) for the anti-Dicer-2 antibody and goat anti-mouse secondary antibody (1:1000; Millipore) for the actin antibody at 26°C. The signal was detected using UVP Chemostudio (Analytik Jena).

### Midgut juice collection

Midgut fluid was collected from second-instar S. litura larvae and adult honey bees. The midguts were dissected under a microscope and homogenized in 10 µL of PBS. The homogenate was centrifuged at 12,000 ×g for 10 min at 4°C, and the supernatant was collected. The midgut sample (1 µL) was diluted with 10 µL of PBS and kept on ice at 4°C until further use. In our experiment, we did not measure the protein concentration of the gut fluid. Instead, we standardized the extraction process by using one midgut per replicate, with each individual larva serving as an independent biological replicate. For the dsRNA incubation assay, 1 µL of midgut fluid was mixed with 1 µL of dsRNA (40 µg) and incubated at the designated time points. The CT values were measured after 30 and 60 minutes to assess dsRNA degradation (21).

### Statistical analysis

Gene expression data were evaluated using one-way analysis of variance (ANOVA), followed by Tukey’s *post-hoc* test to determine significance. Mortality rates were analyzed using the log-rank (Mantel-Cox) test to compare survival distributions. All statistical analyses were performed using GraphPad Prism 9 software (GraphPad Software, USA). Error bars in the graphs represent the standard deviation. Statistical significance was set at a p-value of less than 0.05, as follows: “^*^” for p < 0.05, “^**^” for p < 0.01, and “n.s.” was not significant (13, 16).

## Results

### Enhanced efficacy of siRNA over dsRNA in reducing larval survival of *S. litura*

**Figure 1:**
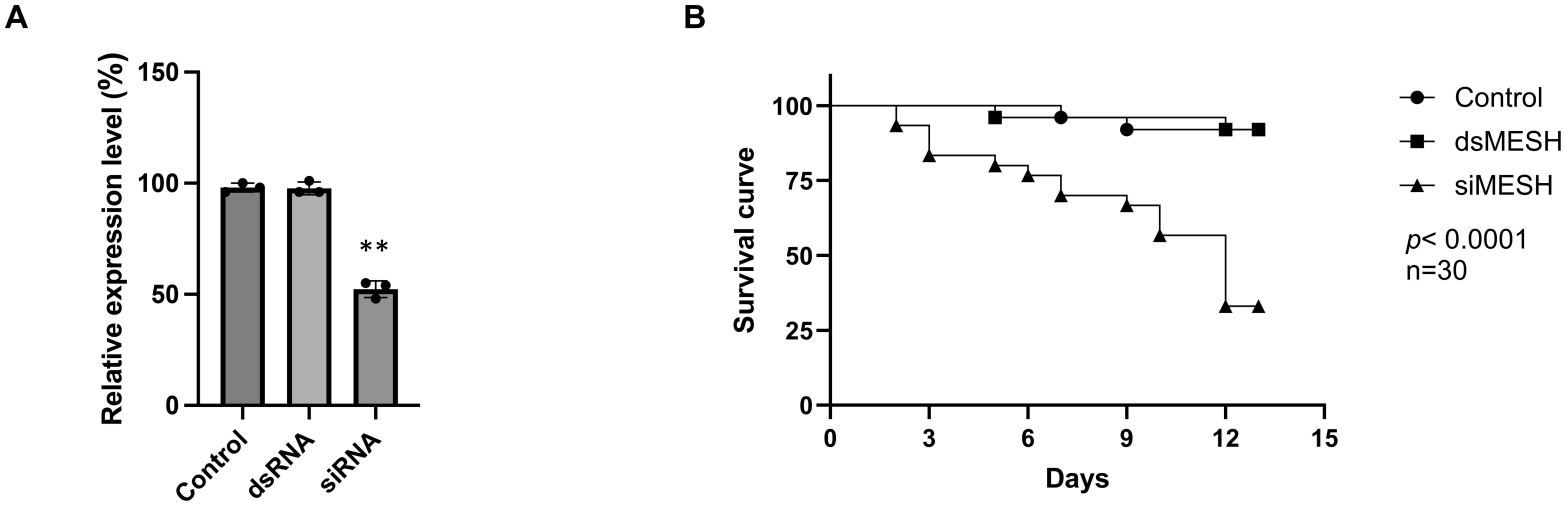
Differential RNAi-mediated silencing of the *mesh* gene and its impact on *Spodoptera litura* larval mortality. (**A**) Expression of the *mesh* gene in the midgut of *Spodoptera litura* larvae 24 hours after feeding with dsRNA or siRNA, measured by qRT-PCR. The control group was fed with siRNA targeting the *egfp* gene. **(B)** Larval mortality assessed 12 days after feeding with 0.3 μg of dsMESH or siMESH. Data represent the mean ± standard deviation from three independent experiments, with 30 larvae per group. Differences between experimental groups were analyzed using Student’s *t*-test. Statistical significance was indicated as, and *p* < 0.01 (**).

**Figure 2:**
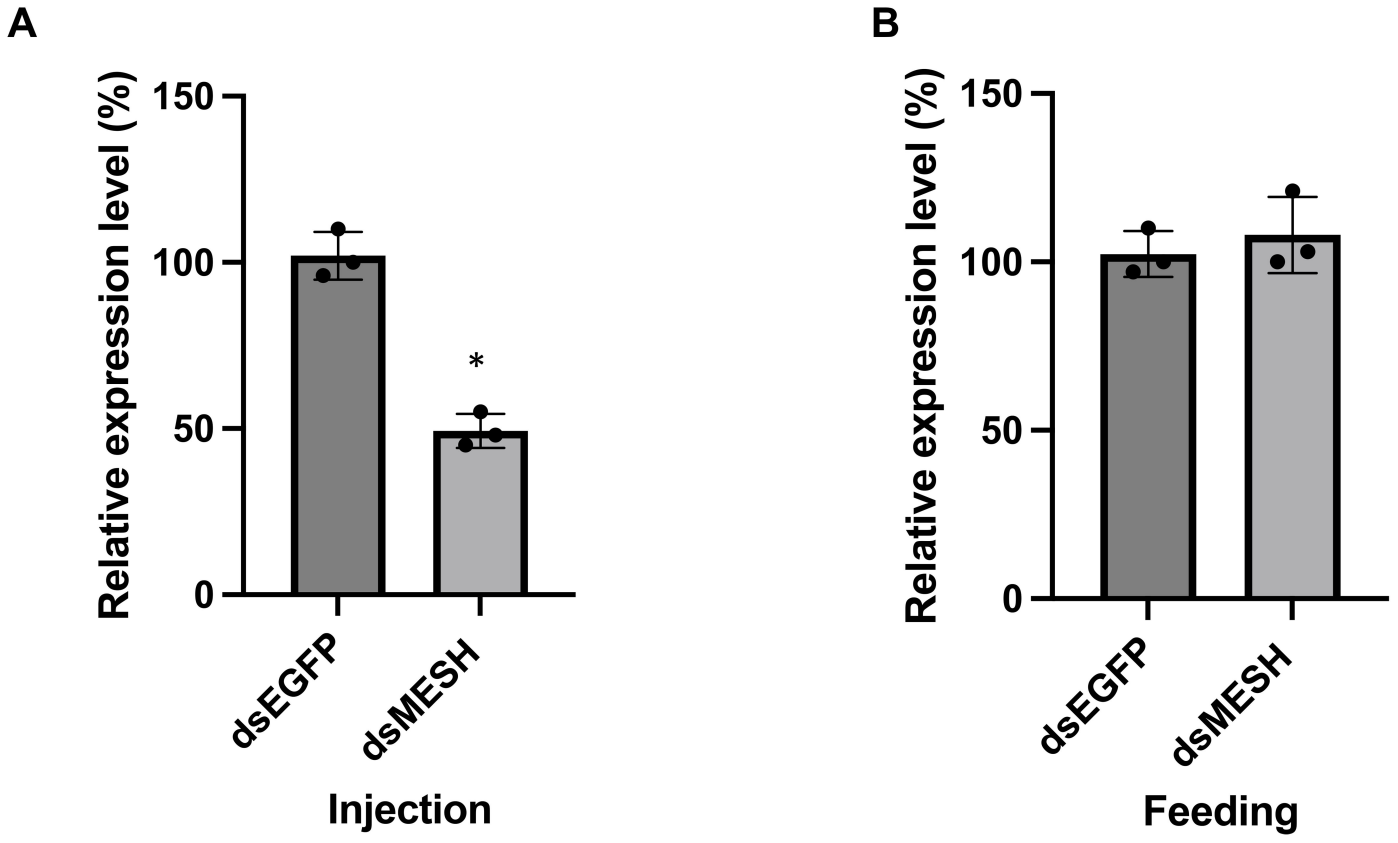
Effect of dsRNA injection and feeding on Spodoptera litura. **(A)** qPCR analysis of *mesh* gene expression 24 hours after injecting 0.3 μg dsRNA per individual. The control group was injected with dsRNA targeting the *egfp* gene. **(B)** qPCR analysis of *mesh* gene expression in the midgut 24 hours after feeding with 0.3 μg dsRNA per individual. Data are shown as mean ± SD. Expression levels in the control group were set to 100%. Three biological replicates were performed. Statistical differences between groups were evaluated using Student’s t-test, with significance defined as p < 0.05 (*).

The feeding of dsRNA and siRNA targeting the *S. litura mesh* gene was compared to assess RNAi efficiency. An aliquot of 0.3 μg per larva of either dsMESH or siMESH was added to the artificial diet (**Figure 1**). qRT-PCR showed that only siMESH significantly suppressed gene expression, with a reduction of approximately 50% after 24 hours of feeding (**Figure 1A**). To further compare insecticidal activity, we assessed mortality after 12 days of dsMESH and siMESH feeding. The results showed that siMESH feeding induced a significantly higher lethal effect compared to dsRNA feeding (**Figure 1B**). Additionally, we injected dsMESH into the haemocoel to determine whether the insufficient RNAi effect was specific to dsMESH feeding. qPCR results showed a significant reduction in mesh expression levels in the dsMESH injection group compared to the dsEGFP injection control (**Figure 2A**), confirming that dsRNA injection effectively induces gene silencing, in contrast to dsMESH feeding (**Figure 2B**).

**Figure 3:**
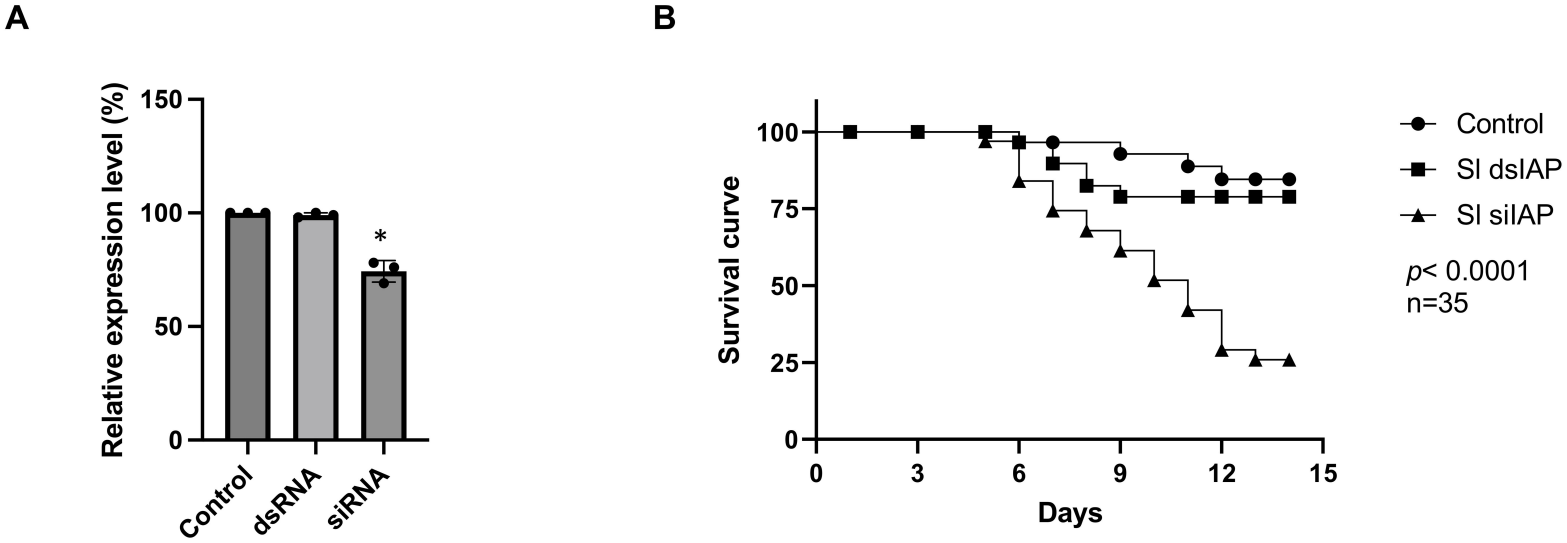
Enhanced insecticidal effect of siRNA Targeting the *iap* Gene in *Spodoptera litura* larvae. **(A)** Expression of the *iap* gene in the midgut of *S. litura* larvae 24 hours after feeding with dsRNA or siRNA, measured by qRT-PCR. The control group was fed with siRNA targeting the *egfp* gene. **(B)** Impact of dsIAP and siIAP feeding on larval survival over 12 days. For all treatments, larvae were fed 0.3 μg of dsRNA or siRNA per individual per day for four consecutive days. While the same mass of dsRNA and siRNA was used, their molar amounts differ due to the shorter length of siRNA, resulting in a higher molar concentration compared to dsRNA. Data represent the mean ± standard deviation from three independent experiments, with 35 larvae per group. Differences between experimental groups were analyzed using Student’s *t*-test. Statistical significance was indicated as *p*< 0.05 (*).

To verify that the difference in RNAi efficiency between dsRNA and siRNA feeding is not gene-specific, we conducted an additional experiment targeting a second gene, the *inhibitor of apoptosis protein* (*iap*). Similar results were observed, showing that siRNA feeding significantly suppressed gene expression (**Figure 3A**) and induced higher mortality (**Figure 3B**) compared to dsRNA feeding.

### Inefficient dsRNA processing and low Dicer-2 expression limit RNAi response in *S. litura*

**Figure 4:**
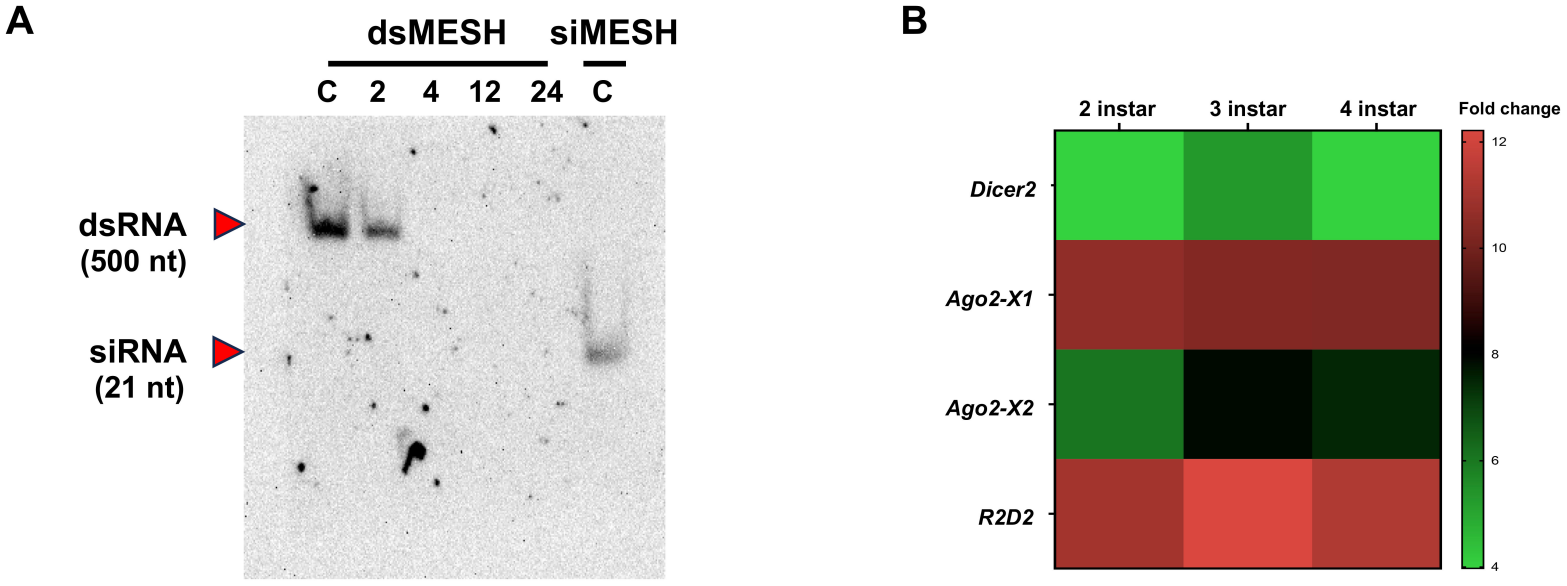
Conversion Efficiency of dsRNA into siRNA and Expression Levels of RNAi-Related Genes in the Midgut of *S. litura*. **(A)** Northern blot analysis showing the conversion of dsRNA into siRNA in *S. litura* larvae. Small RNA was extracted from the midgut at various time points (2, 4, 12 and 24 hours post feeding) following dsRNA ingestion. C: Synthesized small RNA was directly loaded onto a gel as an untreated control. Red arrows represent dsMESH, and red arrows indicate siMESH. **(B)** qRT-PCR analysis of key genes involved in the processing of long dsRNA into siRNA in the midguts of 2nd to 4th instar *S. litura* larvae. *Ago2-X1* and *Ago2-X2* represent alternative splicing isoforms of *Ago2*. The scale bar indicated -ΔΔCT value subtracted by *Dicer2* in each instar. The number in each cell represented the average and standard deviation (n=5).

Given that siRNA feeding induced significant gene silencing and lethality compared to dsRNA feeding, we hypothesized that dsRNA might not be processed into siRNA in the digestive system of *S. litura*. To test this hypothesis, we conducted northern blot analysis of small RNA extracted from midguts at 2, 4, 12, and 24 h after dsMESH feeding, using a DIG-labeled dsMESH probe (**Figure 4A**). The results showed that dsRNA was not converted into siRNA in the midgut at the time points tested. In addition, after dsRNA enters the cell, it is cleaved into siRNA in the cytoplasm by Dicer-2, with the participation of other auxiliary proteins. We used qRT-PCR to analyze the expression levels of genes involved in this process and found that Dicer-2 expression in the midgut of *S. litura* was significantly lower than that of other genes related to siRNA generation, such as *R2D2* and *Ago2*. Notably, *Ago2* was detected in two alternative splicing isoforms, *Ago2-X1* and *Ago2-X2*, with varying expression levels. This suggests potential differences in their functional roles in RNA interference (RNAi) and may contribute to the observed differences in RNAi efficiency (**Figure 4B**).

### Rapid degradation of dsRNA in the midgut fluid and low Dicer-2 expression impedes RNAi function in *S. litura*

**Figure 5:**
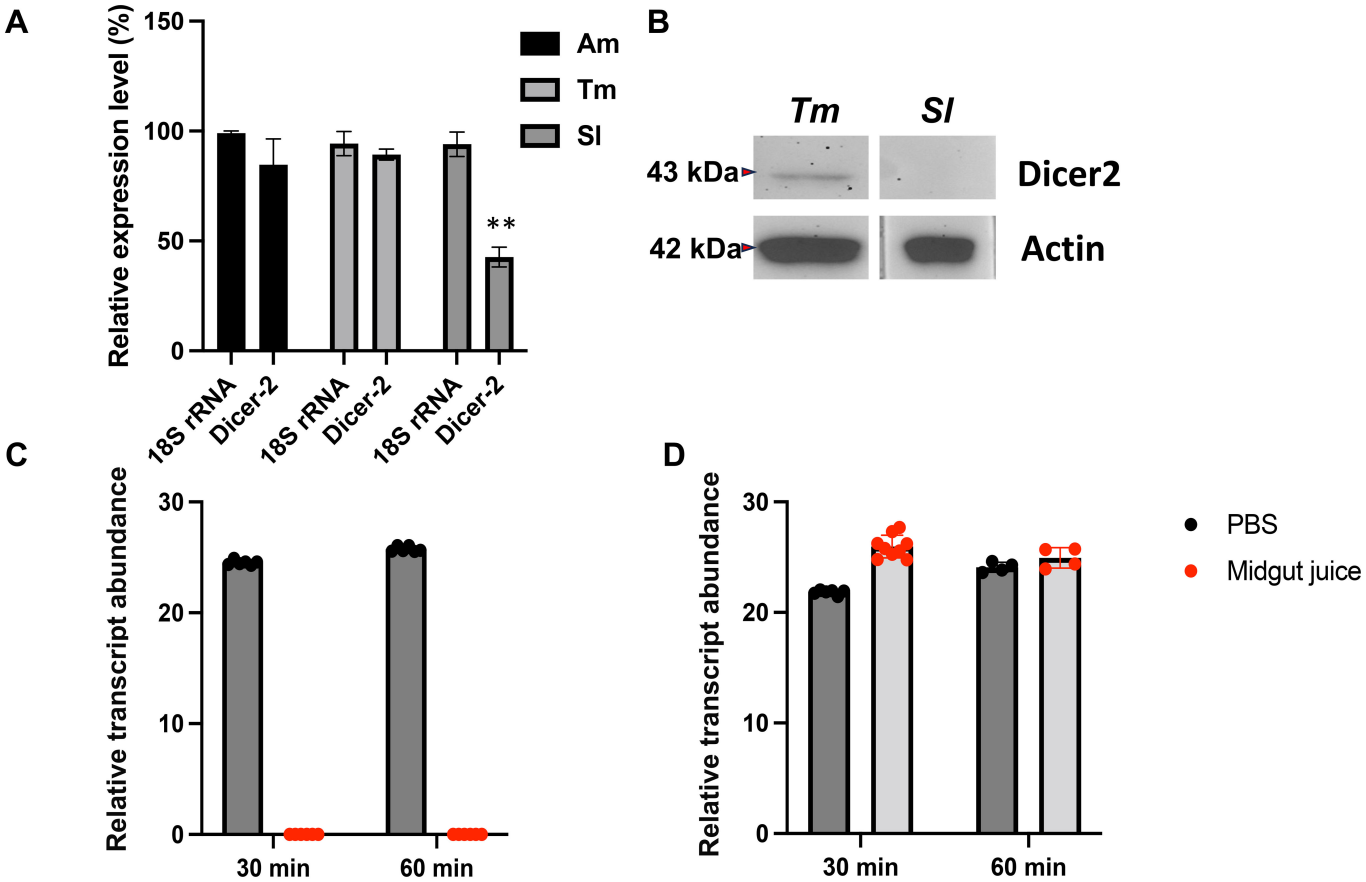
Reduced Dicer-2 expression in the midgut of *Spodoptera litura* larvae impairs the conversion of double-stranded RNA (dsRNA) into small interfering RNA (siRNA). **(A)** qRT-PCR analysis was performed to measure the expression levels of the *dicer-2* gene in the midguts of various insect species, including bees (*Am*), mealworms (*Tm*), and *Spodoptera litura* (*Sl*). The expression level of 18S rRNA was independently quantified for each species and standardized to 100%, serving as a baseline for comparison. The relative expression levels of the *dicer-2* gene were normalized against this baseline. Statistical differences between experimental groups were analyzed using Student’s *t*-test. Statistical significance was indicated as p < 0.01 (**). **(B)** Western blot analysis confirming Dicer-2 expression in the midgut of *S. litura* (Sl) and mealworms (Tm), using anti-Dicer-2 antibodies. Anti-actin antibodies were used as a loading control. Total protein was extracted from the midgut tissue. qRT-PCR analysis of dsRNA degradation in midgut fluid from *S. litura* **(C)** and honey bees **(D)**. Midgut juice was extracted from a single larva per biological replicate, without protein concentration measurement. The gut juice from one larva was mixed with PBS (1:10 dilution) before incubating with 40 μg of dsRNA. Degradation was analyzed at 30 and 60 minutes using qRT-PCR. The y-axis represents the relative transcript abundance, reflecting changes in dsRNA quantity. A relative transcript abundance of 0 indicates undetectable dsRNA levels. The experiment was conducted with four biological replicates.

Using qRT-PCR, we assessed the expression levels of the *dicer-2* gene in the midguts of honeybees (Am), mealworms (Tm), and *S. litura* (Sl), finding that *S. litura* consistently showed significantly lower *dicer-2* expression (**Figure 5A**). This reduced expression was also evident when comparing *dicer-2* levels across various insect species, with *S. litura* displaying the lowest levels. In fact, Dicer-2 protein levels were nearly undetectable in the midguts of *S. litura* (**Figure 5A**). This low *dicer-2* expression may explain the failure of dsRNA to effectively trigger RNAi in the midgut, suggesting that reduced *dicer-2* expression may be an important factor in the inefficient RNAi response.

To explore additional factors affecting dsRNA functionality, we examined dsRNA degradation in the midgut environments of *S. litura* (**Figure 5B**) and bees (Hymenoptera) (**Figure 5B**). In *S. litura*, dsRNA degraded within approximately 20 minutes, indicating strong enzymatic activity that rapidly breaks down dsRNA. By contrast, dsRNA remained stable for up to 60 minutes in bee midgut fluids, reflecting a less aggressive degradation environment (**Figure 5B**). These findings indicate that the failure of dsRNA to function in *S. litura* is due to both low Dicer-2 expression, which limits dsRNA processing into siRNA, and rapid dsRNA degradation in the midgut, which prevents effective gene silencing. Consequently, these physiological limitations suggest that dsRNA-based pesticides are unlikely to be effective against *S. litura.*

## Discussion

**Figure 6:**
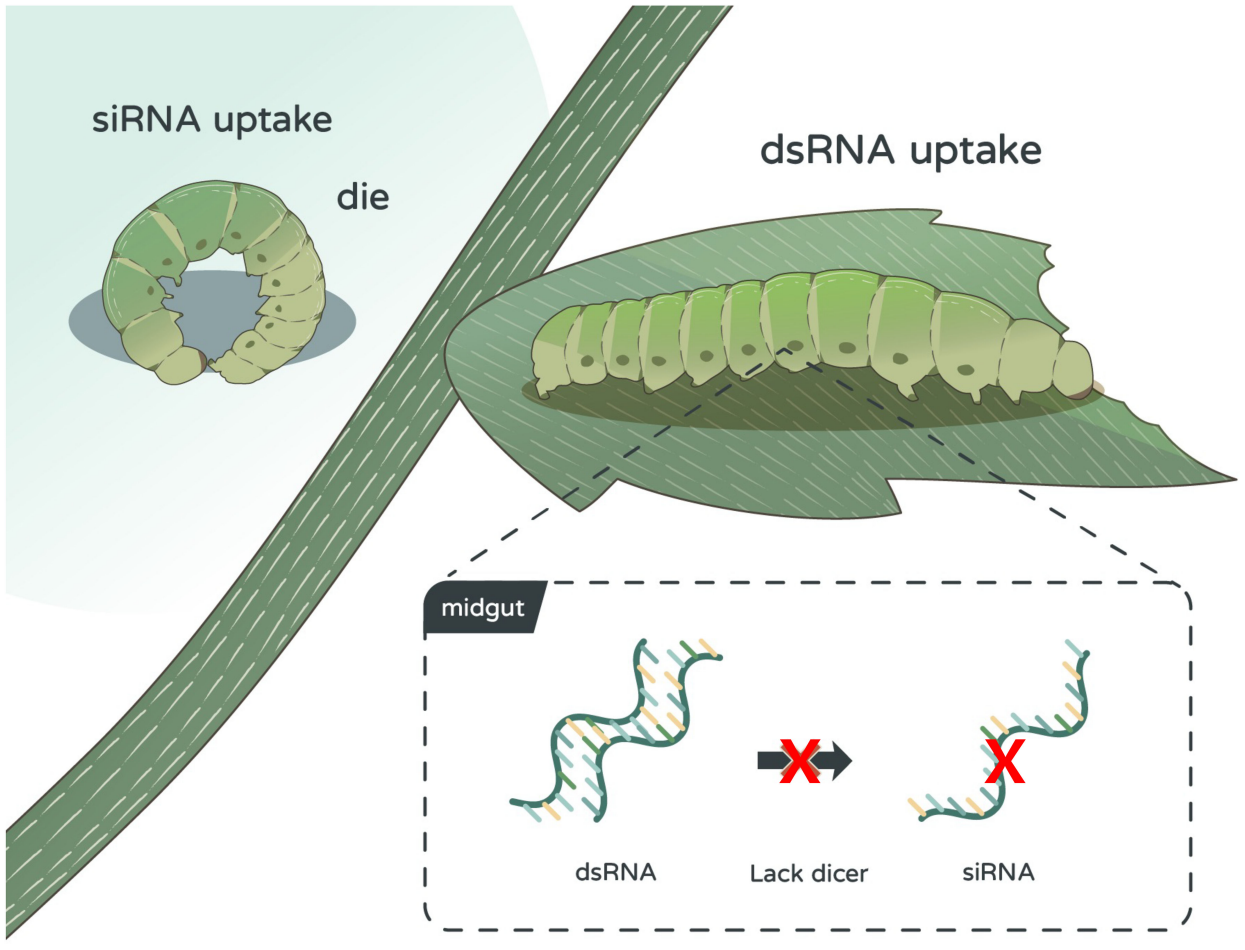
Impact of double-stranded RNA (dsRNA) and small interfering RNA (siRNA) on *Spodoptera litura* larvae. dsRNA shows no impact on *S. litura* larvae, whereas siRNA demonstrates notable toxicity against the larvae. dsRNA’s lack of toxic effect on *S. litura* is attributed to the absence of key genes in the midgut necessary for processing dsRNA into siRNA.

Our study evaluated the effectiveness of RNAi-based pest control using siRNA and dsRNA targeting the *S. litura mesh* or *iap* gene. The observed poor RNAi efficiency of dsRNA treatment aligns with previous reports. Wang et al. (22) noted that chitinase dsRNA was ineffective using both injection and feeding methods, while Rajagopal et al. (23) reported that aminopeptidase dsRNA worked only via injection not feeding (22, 23). While siRNA feeding significantly suppressed gene expression and reduced larval survival in our study, dsRNA showed no such effects. We attribute this to several factors. First, the expression levels of core RNAi pathway components, particularly Dicer-2, were markedly lower in the midguts of *S. litura*, limiting the ability of dsRNA to be processed into functional siRNA (**Figure 6**). This aligns with previous studies, where low expression of Dicer-2 and other core RNAi genes, such as *Ago1*, *Ago2*, and *R2d2*, was observed in *Spodoptera* species, leading to poor responses to dsRNA (24, 25). The lack of effective RNAi machinery in *S. litura* may also explain why dsRNA was not processed efficiently, further supported by our findings that Dicer-2 protein levels were almost undetectable in midgut tissues. The relative expression level (to *18S*) of Dicer-2 in the midgut was significantly higher than *S. litura* (**Figure 5A**). In previous studies, both honey bees and mealworms were demonstrated possessing dsRNA processing abilities and exerted RNAi responses (26, 27). Second, the rapid degradation of dsRNA in the midgut fluid of *S. litura* poses a significant challenge (28). Our degradation assays revealed that dsRNA was broken down within 20 min in *S. litura* midgut fluids. This rapid degradation, coupled with an alkaline gut environment, highlights the enzymatic activity that accelerates dsRNA decomposition, preventing it from exerting its gene-silencing effects. Other factors, such as intestinal pH, also contribute to this degradation, as RNA is more prone to hydrolysis in highly alkaline environments (21). In contrast, siRNA demonstrated higher stability and efficiency in inducing gene silencing, particularly in younger larvae, where cell division and peritrophic membrane synthesis in the midgut were more active. This age-dependent sensitivity suggests that siRNA-based strategies may be more effective against earlier instar larvae, as older larvae showed reduced responsiveness, likely due to the completion of midgut development (25, 29).

Despite these challenges, siRNA exhibited promising results in our laboratory and semi-field trials. Further research should focus on enhancing siRNA stability, particularly through the use of protective carriers or nanoparticles, to increase environmental resilience and improve its efficacy in the field (30). Furthermore, the integration of RNAi-based pesticides into pest management programs will be essential to delay the development of resistance in target pests (31). These findings indicate the limitations of dsRNA in *S. litura* due to inefficient RNAi processing and rapid degradation in the midgut. However, siRNA offers a viable alternative, particularly with the potential for formulation improvements that improve stability and uptake in pest species resistant to traditional RNAi approaches.

## Data Availability

The raw data supporting the conclusions of this article will be made available by the authors, without undue reservation.
